# Targeted Drug Delivery in Periorbital Non-Melanocytic Skin Malignancies

**DOI:** 10.3390/bioengineering11101029

**Published:** 2024-10-15

**Authors:** Benedetta Tirone, Anna Scarabosio, Pier Luigi Surico, Pier Camillo Parodi, Fabiana D’Esposito, Alessandro Avitabile, Caterina Foti, Caterina Gagliano, Marco Zeppieri

**Affiliations:** 1 Dermatology and Venerology Section, Department of Precision and Regenerative Medicine and Ionan Area (DiMePRe-J), University of Bari Aldo Moro, 70124 Bari, Italy; 2 Clinic of Plastic and Reconstructive Surgery, Ospedale Santa Maria della Misericordia, 33100 Udine, Italy; 3 Massachusetts General Hospital, Harvard Medical School, Boston, MA 02114, USA; 4 Schepens Eye Research Institute of Mass Eye and Ear, Harvard Medical School, Boston, MA 02114, USA; 5 Department of Ophthalmology, Campus Bio-Medico University, 00128 Rome, Italy; 6 Imperial College Ophthalmic Research Group (ICORG) Unit, Imperial College, 153-173 Marylebone Rd, London NW15QH, UK; 7 Department of Neurosciences, Reproductive Sciences and Dentistry, University of Naples Federico II, Via Pansini 5, 80131 Napoli, Italy; 8 Eye Clinic Catania San Marco Hospital, Viale Carlo Azeglio Ciampi, 95121 Catania, Italy; 9 Mediterranean Foundation “G.B. Morgagni”, 95125 Catania, Italy; 10 Department of Medicine and Surgery, University of Enna “Kore”, 94100 Enna, Italy; 11 Department of Ophthalmology, University Hospital of Udine, p.le S. Maria della Misericordia 15, 33100 Udine, Italy

**Keywords:** periorbital skin malignancies, targeted drug delivery systems, targeted drugs, basocellular carcinoma, squamocellular carcinoma, Merkel cell carcinoma

## Abstract

Targeted drug delivery has emerged as a transformative approach in the treatment of periorbital skin malignancies, offering the potential for enhanced efficacy and reduced side effects compared to traditional therapies. This review provides a comprehensive overview of targeted therapies in the context of periorbital malignancies, including basal cell carcinoma, squamous cell carcinoma, sebaceous gland carcinoma, and Merkel cell carcinoma. It explores the mechanisms of action for various targeted therapies, such as monoclonal antibodies, small molecule inhibitors, and immunotherapies, and their applications in treating these malignancies. Additionally, this review addresses the management of ocular and periocular side effects associated with these therapies, emphasizing the importance of a multidisciplinary approach to minimize impact and ensure patient adherence. By integrating current findings and discussing emerging trends, this review aims to highlight the advancements in targeted drug delivery and its potential to improve treatment outcomes and quality of life for patients with periorbital skin malignancies.

## 1. Background

**Figure 1:**
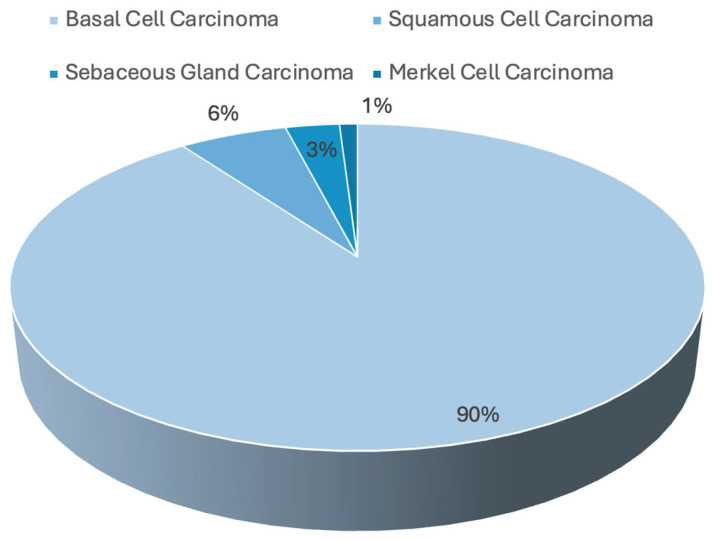
Prevalence of non-melanocytic skin cancers.

Periorbital skin malignancies encompass a wide range of neoplastic conditions that pose unique challenges due to the anatomical and functional complexity of the periocular region. Basal cell carcinoma (BCC), squamous cell carcinoma (SCC), and sebaceous gland carcinoma (SGC) are the most prevalent types of non-melanocytic malignancies in this area [1,2]. Among these, BCC is the most common, representing approximately 90% of periocular malignancies, followed by SCC (Figure 1). These cancers typically present a significant risk for local invasion, given the thin nature of the periorbital skin and proximity to critical structures like the eyelid and orbit, which can lead to significant functional impairment and disfigurement. Traditional treatment approaches, primarily surgical, often lead to significant functional and cosmetic impairments [1,2]. The advent of targeted therapy has revolutionized the management of these malignancies by offering more precise and less invasive treatment options [2]. Targeted therapies, including monoclonal antibodies, small molecule inhibitors, and immunotherapies, specifically attack cancer cells while sparing healthy tissues, thereby reducing side effects and improving outcomes [2,3,4,5,6,7]. In particular, targeted therapies are being explored for periocular malignancies to minimize surgical morbidity, especially in cases in which traditional surgery could compromise vision or cause significant aesthetic damage. The relevance of these therapies is underscored by their growing application in cases in which either surgery is not feasible, or there is a need to reduce tumor burden prior to surgical intervention [4,5].

This review focuses specifically on non-melanocytic malignancies because, while melanoma is a significant concern in periocular oncology, it has been extensively studied in the context of targeted therapies. Numerous reviews and studies have already explored the role of targeted drug delivery in melanoma treatment, including the use of BRAF and MEK inhibitors, and immunotherapies such as checkpoint inhibitors [6]. In contrast, there is a relative lack of research addressing the application of targeted therapies in non-melanocytic periocular malignancies, where a clear gap exists in research and clinical guidance. Our aim is to fill this gap by providing a comprehensive analysis of the current and emerging targeted drug delivery strategies for non-melanocytic skin cancers in the periocular region [7,8,9].

This review aims to provide a comprehensive overview of targeted drug delivery in the treatment of periorbital skin malignancies. It will explore the various types of targeted therapies currently available, discuss their mechanisms of action, and assess their clinical efficacy. Additionally, this review will address the management of side effects associated with these therapies and consider emerging trends and future directions in the field.

## 2. Methodology

This review conducted a comprehensive literature search using PubMed and Reference Citation Analysis (RCA) to gather relevant data on targeted drug delivery in periorbital non-melanocytic skin malignancies. PubMed, managed by the National Library of Medicine, was chosen for its extensive collection of peer-reviewed biomedical research. The search strategy employed keywords and medical subject headings (MeSH) including “periorbital malignancies”, “non-melanocytic skin cancers”, “targeted drug delivery”, “basal cell carcinoma”, “squamous cell carcinoma”, “nanoparticles”, “immunotherapy”, “biodegradable polymers”, and “monoclonal antibodies”. Boolean operators (AND, OR, NOT) were used to refine and ensure the relevance of the search results.

The search focused on English-language articles published up to 2024 to maintain accessibility and relevance to contemporary clinical practices. Titles and abstracts were manually screened, and full texts of pertinent articles were reviewed to extract key information on drug delivery mechanisms, clinical outcomes, advances in nanotechnology, and challenges in targeted therapies for periorbital skin malignancies.

To ensure thoroughness, manual searches of reference lists from included studies and citation tracking were performed to identify additional relevant studies. Articles were selected based on their contribution to the understanding of targeted drug delivery systems, therapeutic efficacy, safety profiles, and recent advancements. This methodology aimed to provide a comprehensive overview of the role and progress of targeted drug delivery in treating periorbital non-melanocytic skin malignancies, with a focus on improving precision, reducing side effects, and optimizing clinical outcomes.

## 3. Classification

Non-melanocytic periorbital skin malignancies are classified based on their cellular origin and histopathological characteristics. Basal cell carcinoma (BCC) is the most common periorbital skin malignancy [2,3,4,5]. It includes subtypes such as nodular, superficial, morpheaform (sclerosing), and pigmented. BCC is known for its slow growth and low metastatic risk, although it can cause significant local tissue destruction if untreated. Squamous cell carcinoma (SCC) is more aggressive than BCC, with a higher propensity for local invasion and metastasis. It often arises from precancerous lesions like actinic keratosis [2,3,4,6,8]. SCC subtypes include keratoacanthoma, intraepidermal (Bowen’s disease), and invasive SCC [9,10]. Sebaceous gland carcinoma is a rare but highly aggressive malignancy, typically originating in the eyelid’s meibomian glands. It presents a high risk of recurrence and metastasis, leading to significant morbidity [9]. Subtypes include papillary, nodular, and pagetoid forms. Merkel cell carcinoma (MCC) is a rare and highly aggressive neuroendocrine tumor that metastasizes early, often resulting in a poor prognosis [11,12,13]. It is characterized by rapid growth. Other rare non-melanocytic malignancies, such as dermatofibrosarcoma protuberans (DFSP) and microcystic adnexal carcinoma (MAC), also occur in the periorbital region, although they are less common [11,12]. Various non-melanocytic skin cancer characteristcs are summarized in Table 1. 

## 4. Mechanisms of Targeted Drug Delivery

Targeted therapy in oncology represents a therapeutic approach that uses drugs designed to target specific molecules involved in the growth, progression, and spread of cancer cells. Unlike traditional chemotherapy, which attacks all rapidly dividing cells, targeted therapy focuses on specific molecular targets, with the goal of reducing side effects and improving treatment efficacy [1,2,3,4]. Targeted therapy acts on specific proteins, receptors, or other molecules that regulate cancer cell growth and survival. Patients often undergo genetic or molecular testing to identify mutations or abnormal expressions of proteins that may be targeted by available drugs [2]. This approach allows treatment to be tailored to the molecular characteristics of the individual patient’s tumor.

These therapies have a variety of strengths, such as increased specificity, reduced side effects, and possible efficacy in tumors resistant to conventional therapies. This treatment modality is the subject of numerous clinical and nonclinical studies aimed at bringing continuous updates in their application and improvements in their limitations [3,6].

Several types of molecular target therapy have been developed in the field of oncology. They can be divided, according to the molecular characteristics of the agents used, into monoclonal antibodies, small molecules, and gene therapy [1]. In recent years, new findings in targeted therapy have been used in targeted drug delivery. The latter is an advanced strategy that aims to deliver drugs specifically into diseased cells or target tissues, minimizing exposure to healthy tissues and reducing side effects [4]. In this regard, nanoparticle-based therapies are becoming increasingly important. While much of the literature on these therapies focuses on broader areas, such as head and neck malignancies, there is a growing need to extend these findings specifically to periorbital malignancies.

## 5. Monoclonal Antibodies

Monoclonal antibodies (mAbs) are defined as antigen-specific antibodies produced in the laboratory by genetic engineering and cell immunology techniques [1]. They are divided into four categories, according to the nature of the chains that they are made from, as murine, chimeric, humanized and human mAbs [5]. According to the category they belong to, the names of these drugs end in -omab, -ximab, -zumab, and -umab [5]. Furthermore, the inclusion of -ci(r)-, li(m)-, or t(u)- indicates that they target the circulatory or immune system or tumor cells, respectively. Moreover, based on the number and characteristics of their ligands, mAbs can be divided into unconjugated mAbs [6], conjugated mAbs [7], and bispecific mAbs [8]. In the first case, they function by themselves, and in the second case, they are linked to another agent (for example, a chemotherapy or radiotherapy agent), and the bispecific mAbs are linked to another mAb [5].

### 5.1. Mechanisms of Action

Unconjugated mAbs have the ability to bind to specific cell surface antigens. In oncology, they exert their function by following various possible mechanisms: binding to an antigen of a tumor cell, agonizing T-response-mediated cells or recognizing the immune system’s antigens [9].

Regarding the mAbs of the first group, they can act by following a direct or indirect mechanism. In the indirect mechanism, the binding to the tumor cell generates the attack on the latter by innate immune system. Destruction of the cell in this case can occur by complement-dependent cytotoxicity (CMC) [10], antibody-dependent cell-mediated cytotoxicity (ADCC) [11,12] or antibody-dependent cellular phagocytosis (ADCP) [13]. In these cases, tumor killing is performed by the recall of complement, natural killer cells, and macrophages, respectively.

In cases in which mAbs act following a direct mechanism, they bind a cell membrane antigen, frequently a receptor, leading to interference with the intracellular signal transduction related to it. Therefore, they may lead to the apoptosis of the tumor cell or disrupt an important pathway in tumorigenesis, or in the ablation of important elements of the tumor microenvironment (TME) [1]. The main targets of this group of mAbs are the epidermal growth factor receptor (EGFR) pathway, Human Epidermal Growth Factor Receptor 2 (HER2/neu), and Vascular Endothelial Growth Factor (VEGF) [5].

The mAbs that agonize the T-cell-mediated response act by recognizing some members of the tumor necrosis factor receptor (TNFR) family, like CD40, OX40, 4-1BB, and GITR [14]. These differ from the mAbs belonging to the third group, which bind to specific molecules of immune system cells by removing the blockage exerted by cancer cells on them. The mAbs of the third group therefore bind to so-called immuno-checkpoint inhibitors (ICIs), and will be addressed later in this study [9]. However, it is necessary to specify that a single monoclonal antibody can activate multiple mechanisms simultaneously. This is the case with trastuzumab, which can both inhibit HER2 and determine ADCC [15]. Conjugated mAbs are bound to a chemotherapeutic drug or radioactive isotope, and bind specifically to tumor cells to release the toxic load directly into the tumor [7]. Bispecific mAbs are designed to recognize two different antigens, allowing for greater specificity or facilitating the recruitment of immune cells, such as T lymphocytes, against the tumor [8].

### 5.2. Applications in Periorbital Skin Malignancies

In the context of periocular skin tumors, the most commonly used mAbs are intracellular pathway inhibitors and ICIs. EGFR-inhibitors have been used in the treatment of cutaneous squamous cell carcinoma (SCC).

EGFR, also known as Her1 or Erbb1, is a tyrosine kinase receptor, which, upon binding to its ligands (epidermal growth factor [EGF] and transforming growth factor-α (TGF-α)) dimerizes and autophosphorylates, leading to the stimulation of cell proliferation, reduction in apoptosis, and blockage of cell differentiation [16]. In particular, this dimerization leads to the activation of multiple possible pathways including RAS/RAF/MEK/MAPK, PI3K/AKT, and STAT [17]. The resultant effect of EGFR activation in both normal and malignant human skin is severe epidermal disorganization and invasion. Inhibitors of the EGFR pathway can act on the receptor (mAbs) or on some intracellular signal elements (small molecules).

#### Squamous Cell Carcinoma

The development of SCC appears to involve the alteration of several intracellular pathways, including tp53, ras, p16/CDKN2A, and EGFR [18]. This results in over-expressions in the cutaneous SCC of the head–neck area, and some studies have proposed EGFR overexpression as an independent predictor of metastasis and correlated it with tumor aggressiveness [19]. Cetuximab is a chimeric monoclonal IgG1 antibody, which acts by the competitive inhibition of EGFR. It was used in a phase II study of metastatic or inoperable whole-body cutaneous SCC with a dosage of 400 mg/m^2^ for induction and a maintenance dose of 250 mg/m^2^. That study reported the achievement of disease control in 69% of the patients [20] and a disease control rate (DCR) at week 6 of 69%. Moreover, cetuximab has been used for the treatment of locally advanced SCC (laSCC) and metastatic cutaneous SCC (mSCC) of the head and neck alone [21] and in combination with radiotherapy, platinum, or placitaxel [22,23,24,25]. Currently, National Comprehensive Cancer Network (NCCN) guidelines contemplate the use of cetuximab in cases of laSCC in combination with radiotherapy (RT) [26], or alone when ICIs are contraindicated or have already failed [27]. This choice stems from the fact that cetuximab has been shown to have a response rate of 28%, a progression-free survival (PFS) of about 4 months, and an overall survival (OS) of about 1 year. These results are significantly lower than those of ICIs, which therefore tend to be preferred over EGFR inhibitors in the management of this malignancy [28]. The literature offers no further important clinical trials concerning the use of EGFR mAb-inhibitors periocularly [29], and in fact, there are no trials underway for its use as neoadjuvant therapy [30].

Despite promising results, cetuximab therapy has several limitations that should be further investigated. Indeed, the rates of relapse and therapeutic failure are likely to be due to early resistance mechanisms developed by the tumor [31]. Among these, the most relevant are increased ligand production, upregulation of EGFR expression, mutations in KRAS, BRAF, NRAS, and PIK3CA genes, and the expression of the EGFR truncation [31].

Panitimumab is a humanized EGFR-inhibitor mAb, and its use in laSCC and mSCC has also been investigated [32]. In a phase II trial conducted on 16 patients, however, it did not return results as encouraging as those of cetuximab. In fact, an overall response rate of 31%, an overall survival of 11 months, and a median PFS of 8 months were reported. The median duration of overall response was 6 months [33].

## 6. Immunotherapy with Checkpoint Inhibitors

ICIs represent a class of mAbs currently used for the immunotherapy treatment of various cancers [34]. It is known that cancer cells develop several mechanisms to ensure their own proliferation, and one of the most important of these is the evasion of immune-surveillance systems [35]. Under physiological conditions, cells of the immune system use fundamental signaling pathways in order to maintain immunotolerance, and the molecules involved are therefore named immune checkpoints [35]. In order to avoid attack by the immune system, tumor cells express molecules capable of stimulating these pathways by ensuring their own growth [36]. Consequently, ICIs interfere with this mechanism, increase immunosurveillance, and impair tumor cell survival.

The main targets of ICIs are expressed by cytotoxic T cells. They are as follows: cytotoxic T-lymphocyte-associated molecule-4 (CTLA-4) [35], programmed cell death receptor-1 (PD-1), and programmed cell death ligand-1 (PD-L1) [37].

### 6.1. Mechanisms of Action

Concerning PD-1 and PDL-1, the first is a receptor present on cytotoxic T cells and the second is its ligand, expressed, in this case, by tumor cells. Their interaction mediates tumor immunoresistance, leading to a series of cellular events, the most important of which are exhaustion of the cytotoxic T cell response and, in addition, the selection of Treg cells able to increase immunosuppression in the TME [38,39]. From a molecular point of view, the interaction between PD-1 and its ligand would appear to result in the phosphorylation of the Lck protein, which recruits the tyrosine phosphatase Shp2, which in turn inactivates TCR and CD28. Their inactivation inhibits the proper functioning of cytotoxic T cells and the related immune system co-stimulation signals [40,41]. Activation of CTLA-4 suppresses the T cell response in the early stages of T cell differentiation and plays a role in T reg cell activation [42].

PD-1, PDL-1, and CTLA-4 inhibitors, therefore, have been shown to lead to the restoration of the normal antitumoral response. They have been used, especially anti-PD-1 and anti-PDL-1, in a variety of tumors, leading to disease control or disease improvement [43].

### 6.2. Applications in Skin Malignancies

Inhibitors of PD-1 and PDL-1 are the most widely used ICIs in the context of periocular skin tumors.

#### 6.2.1. Squamous Cell Carcinoma

**Figure 2:**
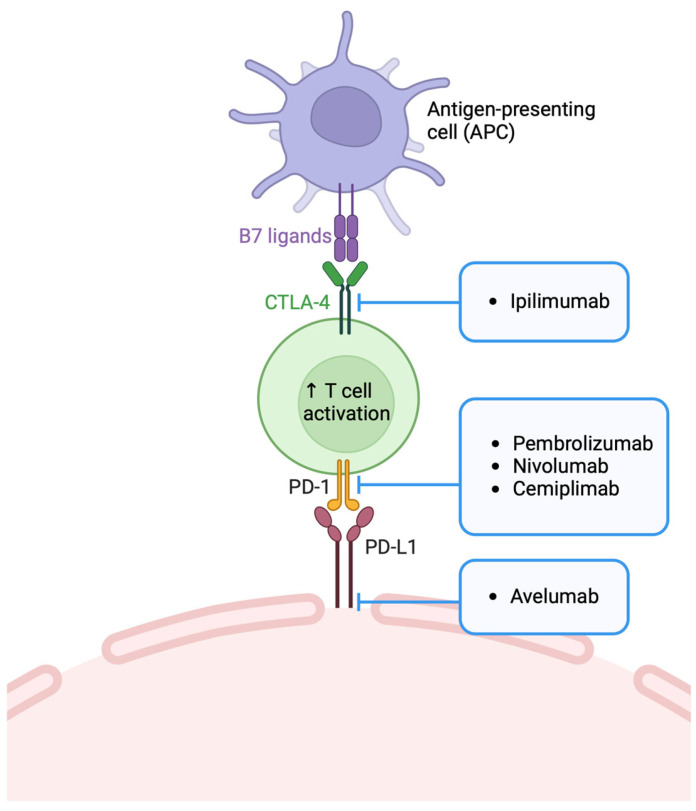
Checkpoint inhibitors’ mechanisms of action.

Cemiplimab and pembrolizumab are anti-PD-1 drugs, which represent one of the systemic therapies currently used in the management of SCC (Figure 2). In this tumor, systemic therapy is used in the case of primary and recurrent locally advanced disease in non-surgical candidates, in patients with regional resected high-risk disease, in patients with unresectable, inoperable or incompletely resected disease, and in patients with regional recurrence or distant metastatic disease [27]. Based on evidence from some important clinical trials, the NCCN guidelines recommend cemiplimab and pembrolizumab in laSCC and mSCC not amenable to surgery and RT, and for use individually. In contrast, chemotherapy and EGFR inhibitors can be combined with RT [27].

There are several data supporting anti-PD-1 in SCC. First, patients treated with ICIs, compared with those treated with other systemic therapies, presented better survival (*P* = 0.034) [44]. This finding could be explained by the high tumor mutation burden that characterizes SCC, which would make it more susceptible to immunotherapy than systemic chemotherapy or targeted therapies [44]. The main adverse events are diarrhoea, fatigue, nausea, constipation, and rash, and a small number of patients discontinue therapy because of side effects. In trials performed on cemiplimab, the most encouraging data were the achievement of a response rate of 54%, complete response (CR) of 13%, and partial response (PR) of 41% [45]. For pembrolizumab, however, the KEYNOTE-629 study reported an objective response rate (ORR) of 50%, CR of 16.7%, and PR of 33.3% [46]. A possible, but not yet fully defined, indication could be the use of anti-PD-1 as neoadjuvant therapy. This application would be particularly important in the setting of periocular tumors to arrive at a more conservative approach. Cemiplimab, used as neoadjuvant, brought a CR of 51% and a major pathologic response of 13% [47]. Pembrolizumab, however, has been less studied in this setting. In small studies conducted on periocular SCC, cemiplimab has led to some encouraging results. In a case series of seven patients with a periorbital SCC, all had a clinically and/or radiologically measurable response as a result of being treated with an anti-PD-1 [48]. There are other case series, in which cemiplimab prevented exenteration in 9/11 [49] and 13/13 [50] patients, respectively. In conclusion, a much larger case history is needed on the use of anti-PD-1 treatments as drugs that can lead to tumor downstaging in a neoadjuvant setting.

#### 6.2.2. Merkel Cell Carcinoma

Merkel cell carcinoma (MCC) is a highly immunogenic tumor in both the polyomavirus-related and unrelated forms [51]. For this reason, immunoresistance is one of the basic tenets of tumor progression, and as a result, immunotherapy has been shown to be extremely effective in its management [52]. Currently, NCCN guidelines recommend the use of ICIs in disseminated MCC as first-line therapy [51], and they are the standard of care even for the treatment of recurrent or inoperable MCC [30]. The currently approved drugs are anti-PDL-1 (avelumab) and anti-PD-1 (pembrolizumab, retifanlimab, and nivolumab) [53]. Figure 2 presents the specific mechanism of action. It would seem that these drugs, in fact, have a response rate comparable to that of chemotherapy (32–64%), with a longer duration of response (DOR) [51]. The use of avelumab is supported by the JAVELIN Merkel 200 trial, in which 116 patients participated and an ORR of 39.7%, a durable response rate (DRR) of 30.2%, and a median overall survival (OS) of 20.3 months were reported [54]. There were also 88 patients treated with as a second-line therapy (after chemotherapy) for whom data up to 49 [55] and 60 months [56] were recorded. The median OS was 12.6 months, and the median OS rates were 31% at 42 months and 28% after 5 years, respectively. The evidence on pembrolizumab came from a phase II study of 50 patients with advanced unresectable MCC that reported an overall response rate of 58% in all the patients and 89.5% for the patients with responsive disease, a 3-year OS of 58.4%, a median PFS of 16.8 months, and a PFS of 39.1% [57]. In the PODIUM-201 study, retifanlimab in 87 patients with advanced (aMCC) or metastatic MCC (mMCC) resulted in an ORR of 46.2% (*n* = 30, 8 CR (12.3%); 22 PR (33.8%)) and a disease control rate (DCR) of 53.8% (*n* = 35) [58]. The efficacy of nivolumab has been proven in various settings. One study evaluated the response of 50 patients with aMCC to therapy with nivolumab alone (*n* = 25) or in combination with ipiliumumab (anti-CTLA-4) (*n* = 25). The difference in ORR in the two groups (72% and 52%, respectively) was not significant [59]. Nivolumab also demonstrated efficacy in a neoadjuvant setting in the CHECKMATE 358 trial. In this phase I/II trial, nivolumab was administered one month before surgery to 36 patients, with a pathologic complete response (pCR) of 47.2%, while 54.5% of the patients had tumor reductions of 30% [60].

The use of ICIs as neoadjuvant treatments could be particularly valuable in the setting of periocular surgery. However, sufficient data on post-surgical outcomes are not yet available. Therefore, neoadjuvant treatment in surgery for MCC (especially periocular) should be discussed with a multidisciplinary team; if the recommendation from the assessment is to opt for this treatment, ICIs, particularly nivolumab, should be preferred over chemotherapy [30]. Concerning the safety of these drugs, avelumab has also been shown to achieve encouraging results in the immunocompromised patient [61]. Avelumab, pembrolizumab, and nivolumab showed treatment-related adverse events (AEs) occurring in 68% to 77% of patients, and grade 3 or 4 AEs occurring in 5% to 21%. Immune-related AEs were seen in less than 20% of patients receiving avelumab, and they were all grade 1 or 2 [53]. Finally, the use of ICIs as adjuvant therapy is currently being studied, but for the time being, the use of chemotherapy or radiotherapy is still preferred in this setting [52].

#### 6.2.3. Basal Cell Carcinoma

ICIs are used as second-line therapy in locally advanced (laBCC) or metastatic BCC (mBC) in patients already treated with HH inhibitors or in whom the latter are contraindicated [62]. Specifically, cemiplimab was investigated with this indication in a study of 84 patients. They were followed with a median follow-up of 15 months, presenting an ORR of 31%. Among these patients, 48% reported grade 3–4 adverse events (AEs) [63].

## 7. Small Molecule Inhibitors

Small molecules are low-molecular-weight molecules (<900 Da) that can penetrate the cell and bind to specific proteins. Thus, they can enhance or inhibit certain cellular pathways, including those implicated in tumorigenesis. There are currently 89 small molecules approved in the US and China in the oncological field [16]. Depending on the target protein, they can act on kinases [64], proteasome, and poly ADPribose polymerase (PARP). By acting on these proteins, they disrupt the signaling pathways involved in carcinogenesis, while in other cases, they promote the apoptosis of tumor cells [1].

### 7.1. Mechanisms of Action

Kinase inhibitors are among the most widely used small molecules in cancer therapy. These enzymes allow the transfer of a group from ATP to a recipient protein. Based on their substrates, protein kinases are classified into tyrosine kinases (receptor-related or not), serine/threonine kinases, and tyrosine-kinase-like enzymes [16]. Proteasomes are multicatalytic enzyme complexes involved in the destruction of misfolded proteins, toxic agents, and other cell-damaging elements. Their inhibition leads to dysfunction and subsequent cancer cell death [65]. PARPs represent a group of post-translational acting enzymes that intervene in various elements of cellular functioning. Most studied has been their role in DNA repair. In this case, the inhibition of the target protein leads to tumor cell death [66]. In addition, there are small molecules directed against proteins implicated in cellular pathways specific to certain tumor types. This is the case of the inhibitors of B-cell lymphoma 2 (BCL-2) family of proteins and the Hedgehog (HH) pathway [16].

### 7.2. Applications in Periorbital Skin Malignancies

Regarding periorbital skin malignancies, they are used in the treatment of Sonidegib (HH-inhibitors) and SCC (EGFR-inhibitors) [30].

#### 7.2.1. Basal Cell Carcinoma

The onset of BCC has been associated with the altered expression of several genes, including the gene for melanocortin 1 receptor (mc1r), oculocutaneous albinism type 2 gene (OCA2), p53, agouti signaling protein (ASIP), and tyrosinase (TYR) [29,67], but the most relevant is the inappropriate activation of the Hedgehog (HH) signaling pathway, which is also present in Gorlin Goltz syndrome or multiple basal cell nevus syndrome. Under normal conditions, Patched-1 (PTCH1), a transmembrane protein, inhibits the smoothened protein (SMO), which, when activated, leads to the activation of the transcription factor Gli and its entry into the nucleus. This event results in the expression of genes involved in the stimulation of cell division. The loss of function of PTCH1, or an increase in the function of the remaining elements of the signaling pathway, therefore favors BCC tumorigenesis. Vismodegib and sonidegib are HH-pathway inhibitors (HPIs) that act by binding the SMO and blocking the entire cellular cascade downstream of that protein. Their use was authorized in 2012 and 2015, respectively, for the treatment of laBCC not eligible for surgery or radiotherapy and mBCC [29,62]. Vismodegib is administered at an oral dosage of 150 mg/day and its long-term efficacy and safety have been reported in several studies. Among these, the ERIVANCE trial [68] and the STEVIE trial [69] should be mentioned. ERIVANCE was a phase II study that evaluated the response to vismodegib in 63 patients with laBCC and 33 patients with mBCC who reported an ORR of 43% and 30%, respectively, in the primary analysis (performed 9 months after the completion of accrual). The long-term update, performed at 39 months, demonstrated ORR values of 60.3% (laBCC) and 48.5% (mBCC), as well as the durability of response, efficacy against aggressive subtypes, and manageable long-term safety of vismodegib in patients with advanced BCC [68]. In the phase II STEVIE trial, a response rate of 68.5% was observed in 1119 patients with laBCC, and a rate of 36.9% was found in 96 patients with mBCC. The trial also confirmed the data on the safety profile and tolerability of the drug [69].

Concerning periocular BCCs, vismodegib could also play a role as neoadjuvant therapy in order to bring about a reduction in tumor size, which would avoid exenteration or surgery that result in the impairment of visual function or in disfiguring outcomes. Several trials have been conducted to support this indication, including VISMONEO [70], VISORB [71], and some previous minor studies [72,73,74]. VISMONEO is a phase II trial aimed at evaluating the ability of vismodegib to reduce the size of the facial BCC in order to opt for less invasive surgery. In total, 55 patients with a facial laBCC, including 19 with a periocular malignancy, were enrolled, and the results were evaluated after 6 months of treatment. Within this population, downstaging was documented in 44% of the cases with a tumor size reduction of 66% and a 70.9% ORR. Moreover, a complete response was registered in 27 patients [70].

In the VISORB trial, 34 patients with periocular laBCC treated with vismodegib for 12 months were examined. Complete response (CR) was reported in 56%, partial response (PR) in 29%, and disease stability (SD) in 26%. In addition, visual function was assessed before and after treatment or surgery following the Visual Assessment Weighted Score (VAWS), and it was found that the patients improved or remained stable in this respect as well. Finally, of the patients who underwent surgery, 67% achieved radicalization [71], and in those with residual disease, an additional SMO mutation was found [75].

Previous smaller studies conducted by Gill et al. [73], Demirci et al. [72], and Ozgur et al. [74] reported similar results about the efficacy of vismodegib in the management of periocular and orbital-invading laBCC.

Furthermore, a trial is currently underway on the 12-week use of vismodegib in patients with a BCC >2 cm [29]. Despite the numerous advantages described, vismodegib is not free of adverse events (AE), which, in some cases have affected the continuity of treatment, namely muscle spasms, alopecia, taste loss, weight loss, decreased appetite, fatigue, nausea, and diarrhoea [32,62].

In conclusion, vismodegib has returned significant results regarding its use as neoadjuvant therapy in laBCC. Such use is particularly important for the management of orbit-invading or periocular BCC, where downstaging of the tumor results in less invasive surgery, which significantly affects the patient’s quality of life [76]. However, several aspects remain to be clarified. Firstly, the duration and treatment regime should be defined, with the possibility of introducing a discontinuous regimen in order to limit the AEs. There is also a need for more long-term follow-up data in order to increase knowledge about the possibility of relapse and resistance and the tolerability of the drug.

Concerning sonidegib, it was studied in the phase II BOLT study. In this study, 230 patients with laBCC and mBCC were observed for 42 months on either a 200 mg or an 800 mg treatment. The data showed ORR values of 56% and 46% in patients with laBCC with a dosage of 200 mg and 800 mg, respectively. The ORR values for mBCC, on the other hand, were 8% and 17%. Furthermore, the disease control rate (DCR) was greater than 80% in all the treated subgroups [77].

These results argue for a possible use of sonidegib for the long-term management of laBCC. Again, most of the patients experienced the side effects already mentioned for vismodegib and, in addition, an increase in creatine kinase [78].

#### 7.2.2. Squamous Cell Carcinoma

**Figure 3:**
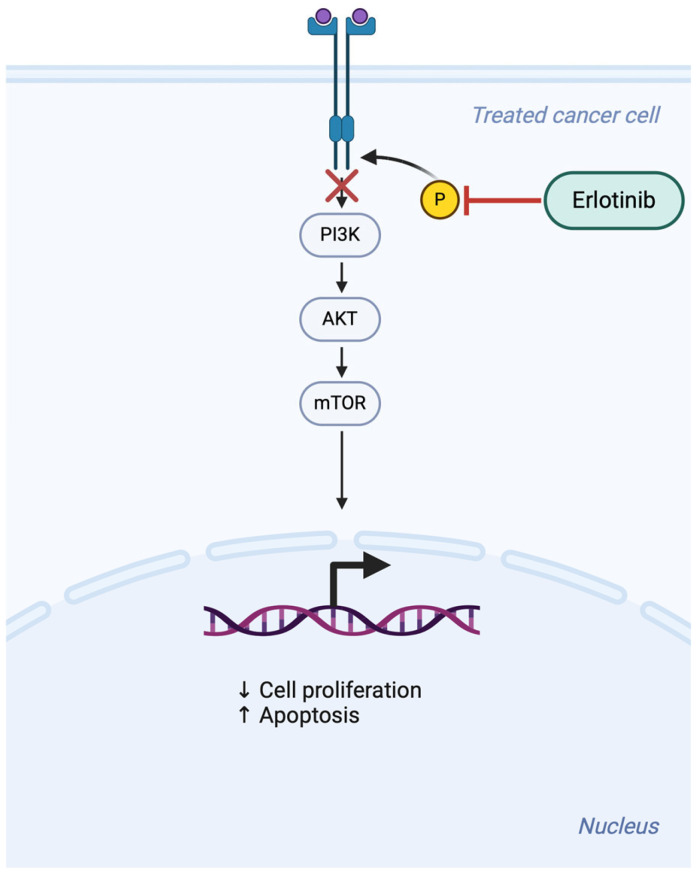
Erlotinib is a small-molecule inhibitor that targets the epidermal growth factor receptor (EGFR), a transmembrane tyrosine kinase receptor involved in cell proliferation and survival. It works by competitively inhibiting the ATP-binding site of EGFR’s tyrosine kinase domain. By blocking EGFR activation, erlotinib prevents the downstream signaling pathways (such as the RAS-RAF-MEK-ERK and PI3K-AKT pathways) that promote tumor cell growth, survival, and proliferation.

In cutaneous SCC, small molecules that inhibit EGFR-activated intracellular signaling (which, we note, is overexpressed in this type of cancer) could be used. To date, some studies have been conducted on the use of two tyrosine kinase inhibitors already used in non-small cell lung carcinoma (NSCLC), namely gefitinib and erlotinib. They act by blocking the autophosphorylation of EGFR and all carcinogenic cellular events related to the latter [16]. Figure 3 presents the specific mechanism of action.

A phase II study of gefitinib as neoadjuvant therapy was conducted in 22 patients with histologically aggressive or recurrent SCC who may or may not have been candidates for surgery. The patients received gefitinib therapy at a dosage of 250 mg/day, and then, depending on response, they were given radiotherapy (preceded or not preceded by surgery) and another 12 months of gefitinib [21,78,79]. The overall response rate to induction therapy was 45.5% Of the four patients with a clinical CR who underwent surgery after induction therapy, three had no residual tumor. In total, 13 of 22 evaluable patients (59.1%) experienced grade 2 toxicities, and the most common side effects were nausea, diarrhoea, fatigue, and acneiform rash [78]. No trials have been performed on the use of erlotinib in SCC. However, there are some cases in the literature in which it has been effectively used in laSCC and mSCC [21,79].

**Figure 4:**
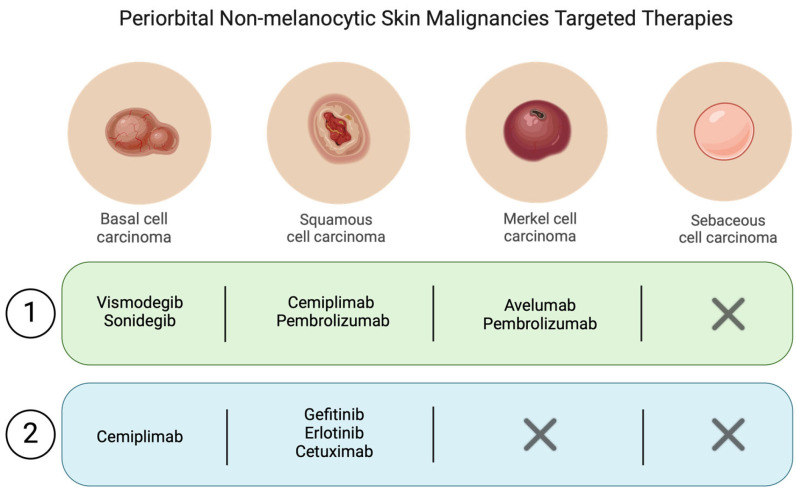
Simplified diagram summarizing the main molecules used for the various non-melanocytic periocular tumors.

In conclusion, based on in vitro studies on and results obtained from the management of other cancers, EGFR inhibitors could lead to promising results for the management of cutaneous laSCC and mSCC. However, larger trials should be performed to obtain more data on ORR, DOR, toxicity, and long-term safety. Figure 4 tries to simplify the actual targeted therapy approach to periocular non-melanocytic skin cancers.

## 8. Other Targeted Therapies

### 8.1. Gene Therapy

Gene therapy provides for the insertion of genetic material (DNA or RNA) inside cancer cells via a vector, leading to the activation or silencing of certain genes. The ultimate goal of this procedure in oncology is cancer cell death or inhibition of disease progression [80].

The gene therapy strategies with application in oncology are oncogene activation inhibition, tumor suppressor gene activation, immunotherapy, suicide gene therapy, and anti-angiogenic gene therapy [81].

The inhibition of an oncogene occurs through the insertion into the cell of an antisense oligonucleotide, which can anneal to cDNA or RNA targets. This results in blocking the transcription of that gene and all elements related to its expression [82].

Usually, these are genes that regulate the cell cycle. Potential targets may be some genes important for tumor cells or for TME, such as, for example EGF, platelet-derived growth factor (PDGF), basic fibroblast growth factor (bFGF), transforming growth factor beta (TGF-β), TGF-α, and VEGF [82].

The activation of tumor suppressor genes (including the Rb gene, the p53 gene, CDK-inhibitors, and BRCA-1/2) allows the expression of the genes involved in cell cycle regulation and apoptosis [81]. For example, gendicine, a recombinant human p53 adenovirus, consists of an adenoviral gene delivery system that is able to insert the p53 gene into cancer cells, thereby stimulating cell death [83].

The application of gene therapy in immunotherapy has been the most thoroughly explored approach until now. It is based on the concept of enhancing the immune-mediated T cell response against tumor cells. To this end, gene therapy allows the development of molecules that are highly specialized in the recognition of tumor antigens, and the most widely used and promising molecules are chimeric antigen receptor (CAR)-T molecules [84]. In CAR-T cell therapy, T lymphocytes from the affected patient are harvested, and a CAR encoding transgene is inserted into them [85]. The clonal expansion of ultra specialized T cells is then stimulated and reinfused into the patient. This technique is currently used in the management of certain lymphomas and leukemias, and in multiple myeloma [81,86].

Regarding antiangiogenic gene therapy, it aims to inhibit tumor neovascularization. An example is the use of a lentivirus gene capable of leading to the production of an anti-VEGF mAb (bevacizumab) in HEK-293 cells [87]. This strategy would achieve a high concentration of bevacizumab and improve its efficacy [81].

In suicide gene therapy, a cytotoxic element is introduced in the form of a prodrug, along with genes encoding enzymes to activate it. In this therapeutic strategy, mesenchymal stem cells could be used as vectors, taking into account their marked ability to migrate into the TME [88]. Currently, the possibility of using the combination of Herpes simplex virus thymidine kinase/ganciclovir (HSV-TK/GC, cytosine deaminase/5-fluorocytosine, cytochrome P450/cyclophosphamide, and carboxypeptidase/4-[2-chloroethyl 2-mesyloxyetel-0-amino] benzoyl-l-glutamic acid) is being explored.

### 8.2. Nanoparticle-Based Delivery Systems

Nanoparticles include liposomes, polymeric nanoparticles, metal oxide nanoparticles, silicon dioxide nanoparticles, and magnetic nanoparticles (MNPs) [89].

In recent years, several innovative strategies have been introduced in the field of cancer therapies, with a focus on increasingly specific and personalized therapy [1]. Nanoparticles represent an effective way to improve the outcomes of drugs available to date, as they can act on their toxicity and selectivity and enhance their mechanisms of efficacy [90]. Nanoparticles fulfil their function in four configurations: association with anticancer agents (nanoparticle-based delivery agents), allowing the combination of multiple anticancer agents (nanoparticle-based codelivery of drugs and genes), allowing the drug to which they are bound to respond to certain chemical/physical stimuli inside or outside the body (stimuli-responsive drug delivery), and association with molecules that recognize certain tumor antigens (nanoparticle-based receptor-targeted delivery) [91].

The nanoparticle-based delivery systems used to date have an active and a passive targeting strategy [92]. Passive targeting corresponds to all the properties of the drug that allow optimization of the drug in reaching tumor cells based on its structural characteristics [93]. Nanocarriers reach the tumor site as a result of enhanced permeability and retention phenomena, known as the EPR effect [94]. Nanoparticles are extremely important in this mechanism because their insertion can change various aspects of drug function. They in fact intervene in pharmacokinetics by increasing drug availability and improve the ability of the drug to diffuse into the TME, thereby increasing uptake by tumor cells and beneficial effects and reducing side effects.

Active targeting makes it possible to reach tumor cells by recognizing and attacking specific antigens [92].

Nanoparticles improve controlled drug delivery and release. They can protect drugs from premature degradation in the body by increasing their stability and circulation times [95].

Alternatively, nanoparticles can be designed to release the drug in response to specific stimuli [89] in the TME, such as acidic pH, the presence of particular enzymes, or even external stimuli, such as heat, light [96], or magnetic fields [97,98]. In addition, nanoparticles are also able to lead to the sensitization of tumor cells. This principle is applied in photothermal and photodynamic therapy [99] and enhanced radiotherapy. Lastly, they can be used as vectors for gene therapy or immune stimulators in immunotherapy [100].

While nanoparticles have been extensively studied in various cancers, their specific application in periorbital malignancies is still in its early stages. Recent studies, however, have begun to explore the potential of nanoparticle-based therapies in this unique anatomical region. For example, polymeric nanoparticles loaded with chemotherapeutic agents have shown promising results in in vitro models of basal cell carcinoma (BCC) and squamous cell carcinoma (SCC), which are the most common non-melanocytic malignancies of the periorbital region [97,98,99,100].

## 9. Side Effects of Targeted Therapies

### 9.1. Ocular and Periocular Side Effects

Targeted therapies, while offering precise treatment options, can also lead to a range of ocular and periocular side effects. These side effects may include conditions such as conjunctivitis, dry eye syndrome, and eyelid inflammation [89]. More severe effects, such as uveitis, keratitis, or optic neuropathy, can also occur, potentially impacting vision and requiring prompt intervention. Hypertrichosis and periocular edema are common and can be particularly distressing for patients due to their impact on appearance. The management of these side effects is crucial to maintaining patients’ quality of life and ensuring continued adherence to treatment regimens [22,28,91,92,93].

### 9.2. Management of Side Effects

The management of ocular and periocular side effects from targeted therapies requires a multidisciplinary approach, often involving collaboration between oncologists and ophthalmologists. Early identification and intervention are key to preventing long-term complications [67,79,86]. Treatment strategies may include the use of lubricating eye drops for dry eye syndrome, corticosteroids for inflammation, and antibiotics for secondary infections. In cases of more severe side effects, such as uveitis or optic neuropathy, discontinuation or adjustment of the targeted therapy may be necessary, along with the introduction of immunosuppressive agents [2,24,86]. Regular monitoring and patient education on recognizing early symptoms are essential to minimizing the impact of these side effects and maintaining adherence to the prescribed cancer treatment.

## 10. Conclusions

In summary, targeted drug delivery represents a significant advancement in the treatment of periorbital skin malignancies, offering the potential for more precise and effective therapies with reduced side effects compared to traditional treatments. By focusing on specific molecular targets, these therapies can improve outcomes and provide new options for patients with malignancies resistant to conventional methods. However, the management of ocular and periocular side effects remains a critical aspect of treatment, requiring vigilant monitoring and a collaborative approach to ensure patient well-being and treatment adherence. As research continues to evolve, the integration of targeted therapies into clinical practice holds promise for further enhancing treatment efficacy and patient quality of life in the realm of periorbital skin malignancies.

## Figures and Tables

**Table 1:** Non-melanocytic periorbital skin malignancies classification.

Malignancy	Common Subtypes	Characteristics
Basal Cell Carcinoma	Nodular, Superficial, Morpheaform (sclerosing), Pigmented	Most common; slow-growing, low metastatic risk; significant local tissue destruction
Squamous Cell Carcinoma	Keratoacanthoma, Intraepidermal (Bowen’s disease), Invasive SCC	More aggressive; higher risk of local invasion and metastasis; arises from precancerous lesions
Sebaceous Gland Carcinoma	Papillary, Nodular, Pagetoid	Rare, highly aggressive; originates in meibomian glands; high risk of recurrence and metastasis
Merkel Cell Carcinoma	N/A	Rare, aggressive neuroendocrine tumor; early metastasis; poor prognosis
Other Rare Malignancies	Dermatofibrosarcoma Protuberans (DFSP), Microcystic Adnexal Carcinoma (MAC)	Less common; includes various rare non-melanocytic tumors
